# UR‐cycleGAN: Denoising full‐body low‐dose PET images using cycle‐consistent Generative Adversarial Networks

**DOI:** 10.1002/acm2.70124

**Published:** 2025-06-02

**Authors:** Yang Liu, ZhiWu Sun, HaoJia Liu

**Affiliations:** ^1^ College of Electronic Information Zhengzhou University of Light Industry Zhengzhou Henan China; ^2^ College of Computer Science and Technology Zhengzhou University of Light Industry Zhengzhou Henan China; ^3^ Department of Medical Imaging The Fifth Affiliated Hospital of Zhengzhou University Zhengzhou Henan China

**Keywords:** deep learning, low‐dose imaging, PET

## Abstract

**Purpose:**

This study aims to develop a CycleGAN based denoising model to enhance the quality of low‐dose PET (LDPET) images, making them as close as possible to standard‐dose PET (SDPET) images.

**Methods:**

Using a Philips Vereos PET/CT system, whole‐body PET images of fluorine‐18 fluorodeoxyglucose (18F‐FDG) were acquired from 37 patients to facilitate the development of the UR‐CycleGAN model. In this model, low‐dose data were simulated by reconstructing PET images with a 30‐s acquisition time, while standard‐dose data were reconstructed from a 2.5‐min acquisition. The network was trained in a supervised manner on 13 210 pairs of PET images, and the quality of the images was objectively evaluated using peak signal‐to‐noise ratio (PSNR) and structural similarity index (SSIM).

**Results:**

Compared to simulated low‐dose data, the denoised PET images generated by our model showed significant improvement, with a clear trend toward SDPET image quality.

**Conclusion:**

The proposed method reduces acquisition time by 80% compared to standard‐dose imaging, while achieving image quality close to SDPET images. It also enhances visual detail fidelity, demonstrating the feasibility and practical utility of the model for significantly reducing imaging time while maintaining high image quality.

## 1 INTRODUCTION

Positron emission tomography (PET) is an advanced medical imaging technology that provides quantitative insights into metabolic and functional activities, critical for clinical diagnostics. Widely used in diagnosing and managing tumors, neurodegenerative diseases, and cardiovascular conditions, PET imaging has established its value in various medical fields. However, the radiation exposure associated with radioactive tracers used in PET remains a notable clinical challenge, particularly for pediatric patients.^1^ Given that children's tissues are still developing, extended radiation exposure significantly raises their risk of developing cancer, emphasizing the necessity of reducing radiation doses while maintaining diagnostic efficacy as a critical research objective in PET imaging.^2^

Reducing tracer dosage in low‐dose PET imaging offers an effective approach to lowering radiation exposure, though it often results in compromised image quality, manifesting as reduced signal‐to‐noise ratio (SNR), blurring, and loss of detail.^3^, ^4^ These quality impairments severely impact the diagnostic reliability of PET images, particularly for detecting small lesions or subtle abnormalities. As a result, balancing image quality with reduced radiation dosage in PET scans has become a major research focus in PET imaging.^5^, ^6^

To mitigate low‐dose PET image degradation, traditional image reconstruction methods have been widely employed. Panin et al. proposed a comprehensive 3D reconstruction method, enhancing the accuracy and noise suppression capabilities of low‐dose PET imaging through improved system matrices.^7^ Similarly, Ahn and Fessler's relaxed ordered subsets algorithm accelerated iterative reconstruction, ensuring global convergence.^8^ Reader and Zaidi provided an in‐depth review of advancements in physics‐based iterative reconstruction, focusing on noise reduction and detail preservation.^9^ However, these methods often compromise image details during noise suppression, potentially affecting diagnostic accuracy. In recent years, with the rapid development of deep learning, Generative Adversarial Networks (GANs)^10^, ^11^ and other neural network models have shown promising results in medical image processing.^12^ Among these, CycleGAN has garnered particular attention in PET image denoising due to its unique capability of training without paired data, making it especially relevant for unsupervised applications.^13^

CycleGAN operates on the principle of learning a mapping between low‐dose and standard‐dose images using dual generators and discriminators without requiring paired training data. By incorporating cycle consistency loss, CycleGAN ensures that a low‐dose input image not only translates into a standard‐dose‐like image but can also be reconstructed back to the original low‐dose image by the reverse generator.^14^ This bidirectional transformation mechanism helps maintain structural information while effectively reducing noise.^15^, ^16^ As a result, images generated by CycleGAN achieve an optimal balance between detail retention and noise removal, a key advantage in the PET denoising domain.^17^

In the broader field of low‐dose CT image denoising, Gu and Ye introduced an adaptive instance normalization (AdaIN)‐based CycleGAN model, effectively denoising low‐dose CT images while preserving anatomical detail by modulating style parameters within the network.^18^ Yang et al. developed a GAN model combined with the Wasserstein distance for low‐dose CT denoising, further enhancing image quality by incorporating perceptual loss.^19^ Chen et al. employed a convolutional neural network (CNN)‐based model for low‐dose CT denoising, which significantly enhanced resolution and detail retention.^20^ In MRI image enhancement, Wang et al. proposed an attention mechanism‐based CycleGAN model, effectively focusing on key regions in the image for improved MRI synthesis.^21^

For PET image restoration and denoising, CycleGAN has demonstrated notable advantages. Cui et al. introduced an enhanced CycleGAN model, IE‐CycleGAN, which improved low‐dose PET image quality by incorporating an advanced cycle consistency loss. This model not only enhanced subjective visual quality but also significantly improved objective metrics, such as peak signal‐to‐noise ratio (PSNR) and structural similarity index (SSIM), making the reconstructed images more suitable for diagnostic purposes.^22^ Amirhossein Sanaat et al. investigated the synthesis of high‐quality images from low‐dose whole‐body PET scans using deep learning algorithms like CycleGAN and ResNet, achieving reduced radiation doses and scan times without compromising diagnostic image quality.^23^ Lei et al. employed CycleGAN for converting low‐statistics PET images into high‐quality, full‐dose‐equivalent images for whole‐body applications.^24^ Additionally, Lei and colleagues further advanced this approach by developing a CT‐assisted CycleGAN network, using CT data to guide PET image reconstruction, substantially improving image quality and accuracy.^25^ These studies highlight the robust potential of CycleGAN in medical imaging and provide innovative solutions for low‐dose PET image reconstruction and denoising.

In conclusion, the application of CycleGAN in low‐dose PET image restoration offers a novel solution for addressing radiation exposure, particularly beneficial for pediatric patients. Through CycleGAN‐based models, PET imaging can achieve reduced radiation dosages while maintaining high‐quality imaging outputs. This development not only aids in protecting patients from excessive radiation risks but also provides a more reliable basis for diagnostic interpretation in medical imaging. As deep learning technologies continue to advance, the future holds promising potential for CycleGAN and its derivative models in enhancing medical imaging applications across diverse domains.

## 2 METHOD

### 2.1 Patients data

This study uses fluorodeoxyglucose (18F‐FDG) body PET images acquired by the Philips PET/CT device, Vereos. There are no restrictions on patient gender, age, or disease type, but cases with artifacts, registration errors, or no obvious lesions were excluded. A total of 37 patient data were included in the study. All patients were intravenously injected with 18F‐FDG at a dose of 3.7 MBq/kg based on their body weight. After the injection, patients rested quietly in the waiting area for 40–90 min before undergoing PET scanning. The scanning was conducted in 3D acquisition mode with a scan time of 2.5 min per bed position. The images were reconstructed using the OSEM algorithm, combining Time of Flight (TOF) and Point Spread Function (PSF), with a matrix size of 288 × 288, two iterations, and six subsets. The slice thickness and slice increment were set to 2 mm. The iteration count for PSF and the regularization factor were set to 1 and 6, respectively. PET attenuation correction was performed using a low‐dose CT scan with 120 kV and 150 mAs.

To simulate low‐dose PET (LDPET) images, a retrospective reconstruction method was applied, shortening the acquisition time to generate LDPET images while maintaining the same reconstruction parameters as for standard‐dose PET (SDPET) images (OSEM 2i6s + PSF + regularization). All PET images were reconstructed using the same parameters. The manufacturer's software handled corrections and image reconstructions, including adjustments for attenuation, scatter, and random coincidences. LDPET images were acquired with a 30‐s acquisition time, while the corresponding SDPET images were reconstructed from 150‐s acquisitions.^26^ The manufacturer‐provided software handled corrections and image reconstructions, including adjustments for attenuation, scatter, and random coincidences. LDPET images were generated with a 30‐s acquisition time, whereas corresponding SDPET images were reconstructed from 150‐s acquisitions.

The dataset was divided into a training set of 13 210 paired PET images, a test set of 864 pairs, and a validation set of 2210 pairs, ensuring rigorous assessment of model performance. Detailed demographic information for the patients included in this study is provided in Table 1. All data were anonymized prior to analysis to protect patient privacy.

**TABLE 1:** Demographics of patients included in this study.

	Training	Test	Validation
Number (patients)	30	2	5
Number (images)	13210	864	2210

### 2.2 Network architecture

#### 2.2.1 CycleGAN

**FIGURE 1:**
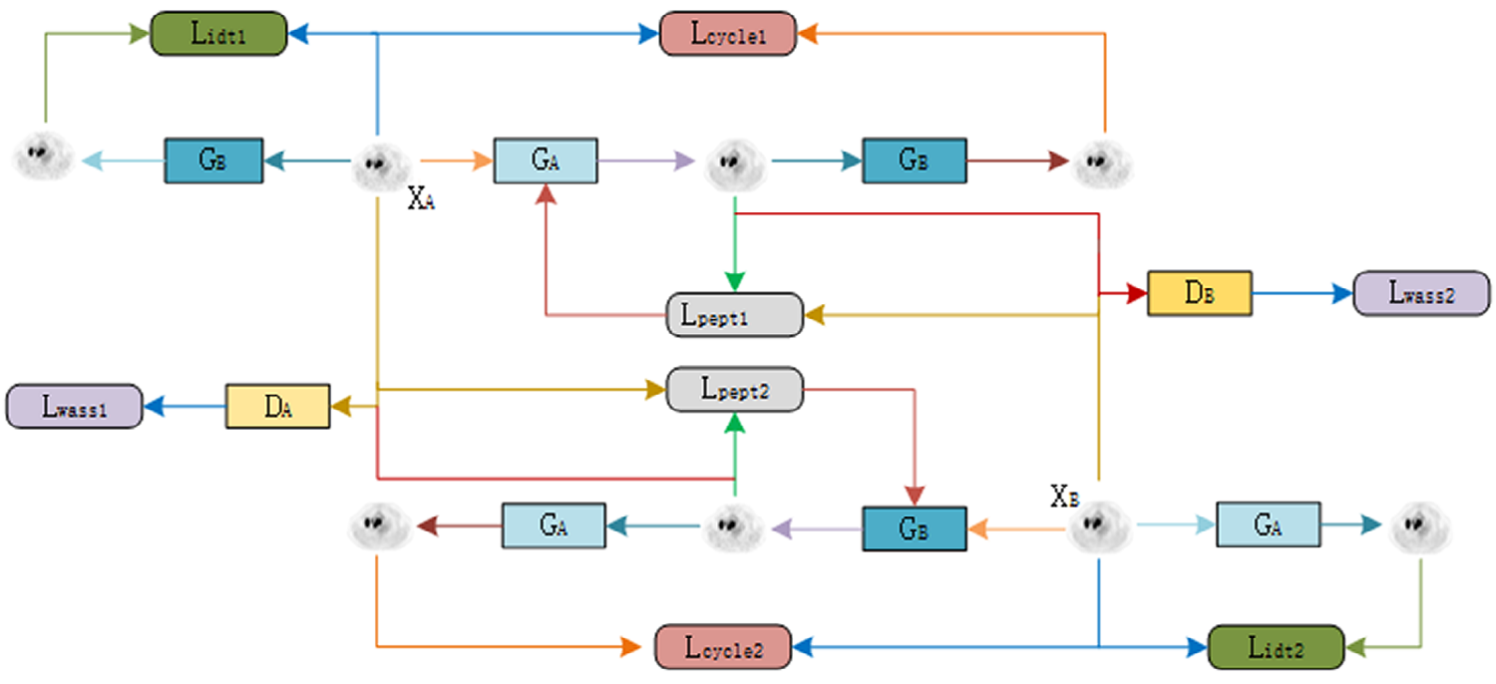
The denoising network process for LDPET images.

In this section, we describe the architecture of our proposed model, UR‐cycleWGAN, which effectively transfers images from domain A to domain B without requiring paired training datasets. As illustrated in Figure 1, the framework consists of two symmetrical GAN networks, utilizing shared generators and discriminators to achieve the mapping between the two image domains. We define domains A and B as LDPET and FDPET images,^27^ respectively. The generator G_A_ represents the mapping from A to B, while G_B_ denotes the mapping from B to A. The discriminators D_A_ and D_B_ are employed to determine whether the input images are generated by the respective generators or are real images.

The specific workflow is as follows (using the upper half as an example):

1. Input image X_A_(LDPET) is processed through generator G_B_ to obtain G_B_(X_A_), and we compute the Identity Loss between X_A_ and G_B_(X_A_).
2. Input image X_A_(LDPET) is processed through generator G_A_ to obtain G_A_(X_A_), and we compute the perceptual loss between X_B_(FDPET) and G_A_(X_A_).
3. Input image X_B_(FDPET) is processed through generator G_B_ to obtain G_B_(X_B_), and we calculate the adversarial loss between G_B_(X_B_) and X_A_(LDPET)
4. Input image X_A_(LDPET) is processed through generator G_A_ and then through generator G_B_ to obtain G_B_(G_A_(X_A_)), and we compute the cycle consistency loss between G_B_(G_A_(X_A_)) and X_A_.

The total network loss is defined as the combination of these losses, ensuring accurate translation and preserving the integrity of the images across the two domains.

(1) $$L=\lambda_{\mathrm{idt}}\;L_{\mathrm{idt}}+\lambda_{\mathrm{cycle}}L_{\mathrm{cycle}}+L_{\mathrm{adv}}+\lambda_{\mathrm{pept}}L_{\mathrm{pept}}$$

#### 2.2.2 Generator

**FIGURE 2:**
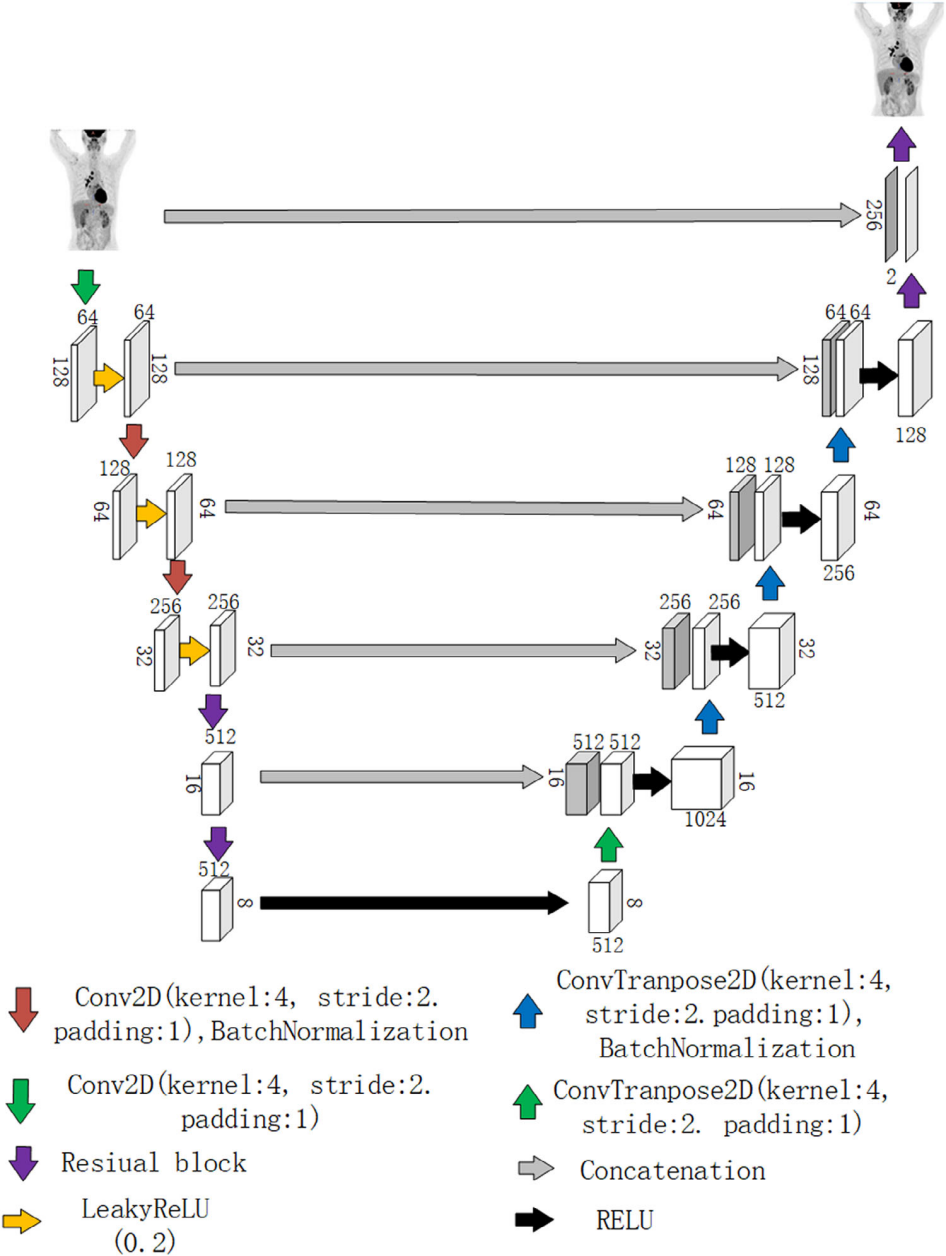
Generator network architecture.

The generator network proposed in this paper is based on the UNet architecture, incorporating residual blocks to enhance the generative capabilities of the model.^28^ as shown in Figure 2. The input to the network is a single‐channel image (256 × 256 × 1), which undergoes five downsampling and six upsampling operations to produce the reconstructed image. During the downsampling process, the network employs multiple layers with skip connections, each consisting of convolution operations, normalization (either Batch Normalization or Instance Normalization), and Leaky ReLU activation functions. These skip connections enable the direct transmission of input features to the corresponding upsampling layers, allowing for the preservation of more detailed information. In the upsampling phase, the network progressively restores spatial resolution through transposed convolution operations. To further optimize image quality, several residual blocks are introduced after multiple skip connections, with each residual block consisting of two convolution layers activated by ReLU. Compared to the traditional UNet architecture, our generator network incorporates five downsampling operations, resulting in a feature map size of 8 × 8 after the final downsampling. Additionally, residual blocks are used to replace some of the standard convolution layers.

#### 2.2.3 Discriminator

**FIGURE 3:**
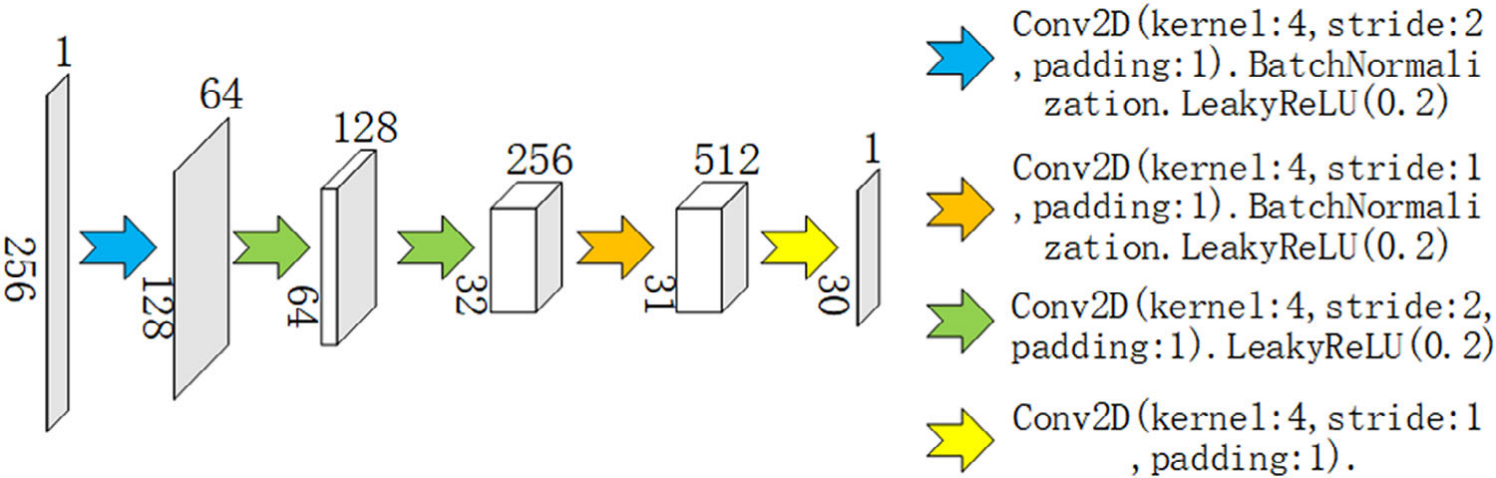
Discriminator network architecture.

The discriminator employs a PatchGAN architecture to assess the authenticity of generated images^29^ as shown in Figure 3. The input to the discriminator is a single‐channel image (256 × 256 × 1), which is processed through multiple convolutional layers to progressively reduce spatial dimensions and extract high‐dimensional features from the image. To further enhance the discriminator's ability to evaluate generated images, we designed a multi‐scale discriminator composed of three sub‐discriminators operating at different scales.^30^ Each sub‐discriminator utilizes spectral normalization techniques to stabilize the training process and prevent instability caused by excessive gradients.^31^ By applying average pooling for downsampling on the input image, the sub‐discriminators can extract multi‐scale features, allowing the overall discriminator to evaluate the generated images comprehensively across different scales, capturing more details and contextual information. Additionally, spectral normalization constrains the spectral norm of the convolutional kernels, limiting the maximum singular value of the weight matrix, thereby ensuring the stability of the discriminator's output and enhancing the model's robustness during training. This multi‐scale discriminator structure effectively improves the discriminator's capability to assess authenticity at various scales, enabling a more detailed evaluation of generated images while enhancing training stability and convergence performance.

#### 2.2.4 Loss function

##### Perceptual loss function

The basic CycleGAN network uses three types of loss functions: adversarial loss (L_advantage), cycle consistency loss (L_cycle), and identity loss (L_identity). To enhance the denoising effect of the network, we introduced perceptual loss based on the original architecture.^32^ The perceptual loss aims to encourage the generator to preserve the details and information content of the images while recovering low‐dose images to standard‐dose images. To achieve this, we utilized the VGG‐19 network as a feature extractor.^33^ VGG‐19 consists of 16 convolutional layers and three fully connected layers, from which we extract feature maps at the output of the 16th convolutional layer. By calculating the differences between the generated images and the target images in the feature space, perceptual loss serves as a guiding signal for the generator, prompting it to produce images that are closer to the target images. In our implementation, the formula for perceptual loss is as follows:

(2) $$L_{\mathrm{pept}}=\frac{1}{N}\;\sum_{i=1}^{N}∥\phi \left(G\left(x_{i}\right)\right)-\phi \left(y_{i}\right){∥}_{1}$$

where N is the batch size, ϕ represents the pre‐trained VGG19 network, G(x_i_) and y_i_ are the generated image and the real image, respectively.

##### Advantage loss (Wasserstein distance)

The original GAN network uses Mean Squared Error (MSE) as the objective function for adversarial loss, but this approach can lead to slow convergence and may result in mode collapse. To overcome these issues, we replaced MSE with Wasserstein distance.^34^ Wasserstein distance significantly alleviates the stability problems associated with using Jensen‐Shannon divergence to compare data distributions, thereby improving the convergence and generation quality of GANs. In our implementation, the adversarial loss function employs the Wasserstein distance formula with a gradient penalty term.^35^

(3) $$\begin{array}{cc} & \min_{G}\;\max_{D}\;L_{\text{WGAN-GP}}\left(D,G\right)\\ & =E_{x∼P_{r}}\left[D\left(x\right)\right]-E_{z∼P_{g}}\left[D\left(G\left(z\right)\right)\right]\\ & +\lambda E_{\hat{x}}\left[{\left(∥\nabla_{\hat{x}}D\left(\hat{x}\right){∥}_{2}-1\right)}^{2}\right] \end{array}$$

x represents samples drawn from the real data distribution Pr, and z denotes samples drawn from the prior noise distribution. The first two terms estimate the Wasserstein distance, while the last term is the gradient penalty for network regularization. λ is the weight hyperparameter for the gradient penalty term.

##### Cycle consistency loss

The purpose of cycle consistency loss is to ensure the mutual convertibility between LDPET and FDPET. Specifically, this loss function guarantees that an input image, after being processed by both generators, is as close as possible to the original image. This mechanism promotes consistency and stability in the network's performance during image transformation tasks. Cycle consistency loss is composed of two components: forward cycle consistency loss and backward cycle consistency loss. The formula is as follows:

(4) $$\begin{array}{cc} L_{\mathrm{cycle}} & =\frac{1}{N}\;\sum_{i=1}^{N}\left(∥G_{B}\left(G_{A}\left(X_{A}\right)\right)\right.\\ & \;\left.-X_{A}{∥}_{1}+∥G_{A}\left(G_{B}\left(X_{B}\right)\right)-X_{B}{∥}_{1}\right) \end{array}$$

The first term is the forward cycle consistency loss term, $G_{B}\left(G_{A}\left(x_{A}\right)\right)\approx x_{A}$;the last term is the backward cycle consistency loss term, $G_{A}\left(G_{B}\left(x_{B}\right)\right)\approx x_{B}$

##### Identity loss

The primary role of identity loss is to enhance the authenticity and stability of the generative model, ensuring that when an input image is already in the target domain, the generator does not make unnecessary modifications to the image. Specifically, when the original data are input into the generator, the generated image should be consistent with the input image, that is, G(x) ≈ x. This mechanism not only ensures the robustness of the model but also effectively avoids unnecessary image transformations, thereby enhancing the stability of the generated results.

(5) $$L_{\mathrm{idt}}=\frac{1}{N}\;\sum_{i=1}^{N}\left(∥G_{A}\left(X_{B}\right)-X_{B}{∥}_{1}+∥G_{B}\left(X_{A}\right)-X_{A}{∥}_{1}\right)$$

### 2.3 Objective image analysis

We use PSNR and SSIM between the estimated standard‐dose image and the true standard‐dose image as metrics to assess image quality.^36^

(6) $$PSNR=20*log_{10}\left(\frac{MAX}{MSE}\right)$$

where MAX represents the peak intensity of the standard‐dose image, and MSE is the mean squared error between the standard‐dose and low‐dose images.

(7) $$\mathrm{SSIM}\;=\frac{2\mu_{x}\mu_{y}+C_{1}}{\mu_{x}^{2}+\mu_{y}^{2}+C_{2}}\;*\;\frac{2\sigma_{\mathrm{xy}}+C_{2}}{\sigma_{x}^{2}+\sigma_{y}^{2}+C_{2}}=\;l\left(i,j\right)*\mathrm{cs}\left(i,j\right)$$

where μx and μy are the mean values of two image patches, σx and σy represent their variances, and σxy is the covariance between the two patches. This metric combines luminance similarity l(i,j) and contrast‐structure similarity cs(i,j), providing a comprehensive assessment of visual similarity between the two images.

### 2.4 Training details

During the training phase, the Adam optimizer was employed along with the Cosine Annealing Warm Restarts (CAWR) learning rate scheduler. The design principle of CAWR is to enhance the training performance of the model by periodically restarting the learning rate.^37^ In this study, the parameters were set to T_0 _= 10 and T_mult _= 2. This configuration allows the learning rate to experience rapid decay during the initial cycles, followed by an increasing cycle length upon each restart. This approach enables the model to converge quickly in the early stages of training while maintaining an appropriate learning rate range in the later stages, facilitating better exploration of the loss landscape and improving generalization performance. For the UR‐CycleWGAN denoising model, a patch size of 32 was set, and the weight hyperparameter for the gradient penalty term, λ was fixed at 10. The selection of these hyperparameters was based on experimental outcomes, conducted over a total of 60 cycles. The learning rate was set to 1e−4, with the momentum estimates' exponential decay rates set to β1 = 0.5 and β2 = 0.999. Each training session for the UR‐CycleWGAN network lasted 10 h, with each cycle requiring 10 min to complete. The experiments were conducted on a computer equipped with an NVIDIA GeForce RTX 4090 GPU.

## 3 RESULTS

### 3.1 SUV performance

SUV, as a commonly used metric for assessing PET uptake, plays a crucial role in clinical diagnosis. In this experiment, lesions and the liver were selected as regions of interest (ROIs) for SUV calculation. Initially, the ROIs were delineated on standard‐dose PET images and then transferred to low‐dose PET images and enhanced PET images for analysis. To further investigate, SUVmax, SUVmean, and standard deviation (SD) were calculated. Measurements for the six datasets (Low‐dose, standard‐dose, RED‐CNN, U‐cycleGAN, U‐cycleWGAN, and UR‐cycleWGAN) were individually computed based on the cases shown in Figure 5. For the U‐cycleGAN, U‐cycleWGAN, and UR‐cycleWGAN datasets, the corresponding values were the average SUV values from three images with different downsampling frequencies, taken from the lesion and liver areas. The lesion region was defined as a 3 cm^2^ area on the transverse image, ensuring that the drawn region did not include surrounding normal tissues. For specific details, please refer to Table 2.

**TABLE 2:** Objective image analysis statistics.

	Low‐dose	Standard‐dose	RED‐CNN
Liver SUV MAX	3.648	3.493	3.984
Liver SUV MEAN	2.741	2.542	2.611
Lesion SUV MAX	9.165	10.251	9.244
Lesion SUV MEAN	5.659	6.469	5.552
	U‐cycleGAN	U‐cycleWGAN	UR‐cycleWGAN
Liver SUV MAX	3.639	3.587	3.573
Liver SUV MEAN	2.624	2.582	2.546
Lesion SUV MAX	9.864	9.326	9.427
Lesion SUV MEAN	5.832	5.866	5.934
	Low‐dose data (Mean ± SD)	Generated data (Mean ± SD)	Standard‐dose data (Mean ± SD)
Liver SUV MAX	3.1516 ± 0.63	3.0207 ± 0.6143	2.8879 ± 0.6215​
Liver SUV MEAN	2.521 ± 0.5316	2.5113 ± 0.5427	2.4633 ± 0.4848
Lesion SUV MAX	13.2052 ± 5.8883	13.1332 ± 6.5324	13.4728 ± 6.2388
Lesion SUV MEAN	7.7796 ± 2.1178	7.6654 ± 2.2651	8.3654 ± 2.2457

*Note*: Low‐dose: Low 18F‐FDG dosage; RED‐CNN: Low 18F‐FDG dosage with enhanced image quality by deep learning method; Standard‐dose: Standard 18F‐FDG dosage; U‐cycleGAN: U‐cycleGAN uses the Unet network as the generator and employs MSE as the adversarial loss function for the network; U‐cycleWGAN: Based on U‐cycleGAN, Wasserstein distance replaces MSE as the adversarial loss function, and perceptual loss is introduced; UR‐cycleWGAN: Based on U‐cycleWGAN, residual blocks are incorporated into the network.

### 3.2 Subjective image analysis

In the initial stage of our experiments, we trained a CycleGAN model using MSE as the loss function. While maintaining consistent hyperparameters, we modified the loss function and network architecture, testing with four, five, and six downsampling operations. The resulting PSNR values for each setting were 30.266, 30.751, and 29.892, and the SSIM values were 0.9180, 0.9218, and 0.9289, respectively, as shown in Figure 6. Notably, the original low‐dose image had a PSNR of 30.738 and an SSIM of 0.9141, indicating limited enhancement in image quality from these adjustments.

To visually evaluate the effectiveness of our proposed approach, we selected a representative PET slice for qualitative comparison, as illustrated in Figures 7 and 8. By comparing the different sections (a), (b), and (c) in Figure 6, the improvement in image quality is apparent. Figure 5a,c represent the corresponding standard‐dose and simulated low‐dose images referenced in Figures 6, 7, 8. Detailed quantitative metrics are presented in Table 3, while subjective visual assessments are provided in Figures 5, 6, 7, 8.

**TABLE 3:** Comparison of image quality evaluation metrics for U‐cycleGAN, U‐cycleWGAN, and UR‐cycleWGAN at different downsampling levels.

		Figures 6, 7, 8		T E S T	
	Structure	PSNR	SSIM	PSNR(mean ± range)	SSIM (mean ± range)
LDPET		30.738	0.9141	31.054 ± 18.703	0.9294 ± 0.1242
RED‐CNN		31.896	0.9236	31.741 ± 19.200	0.9366 ± 0.1205
U‐cycleGAN	16*16	30.266	0.9180	31.281 ± 19.322	0.9325 ± 0.1216
	8*8	30.751	0.9218	31.36 ± 19.241	0.9359 ± 0.1220
	4*4	29.892	0.9289	31.267 ± 18.660	0.9375 ± 0.1419
U‐cycleWGAN	16*16	32.283	0.9196	31.888 ± 18.441	0.9348 ± 0.1530
	8*8	32.889	0.9245	31.89 ± 19.197	0.9378 ± 0.1482
	4*4	31.298	0.9238	31.381 ± 18.982	0.9389 ± 0.1439
UR‐cycleWGAN	16*16	33.792	0.9331	32.487 ± 19.324	0.9409 ± 0.1449
	8*8	**34.403**	**0.9377**	33.455 ± 18.676	0.9436 ± 0.1447
	4*4	33.465	0.9301	32.346 ± 17.644	0.9402 ± 0.1644

*Notes*: Structure: The size of the feature map in the final layer after downsampling in the network is as follows: 16 × 16 indicates the network has undergone four downsampling operations; 8 × 8 indicates the network has undergone five downsampling operations; 4 × 4 indicates the network has undergone six downsampling operations.

The numerical form used in the table is mean ± range, where range = max value—min value.

**FIGURE 4:**
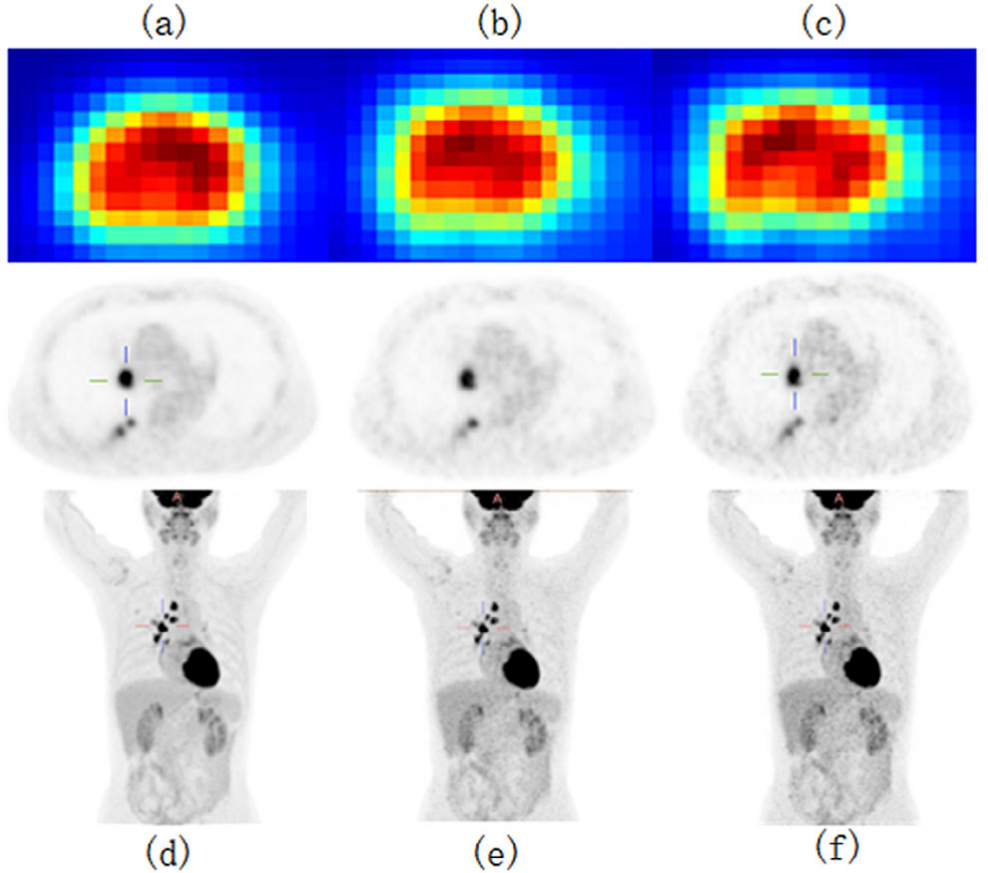
Visualization of standard‐dose images (a, d), Restored images that were generated using our method on the low‐dose images (b, e), Visualization of low‐dose images (c, f).

**FIGURE 5:**
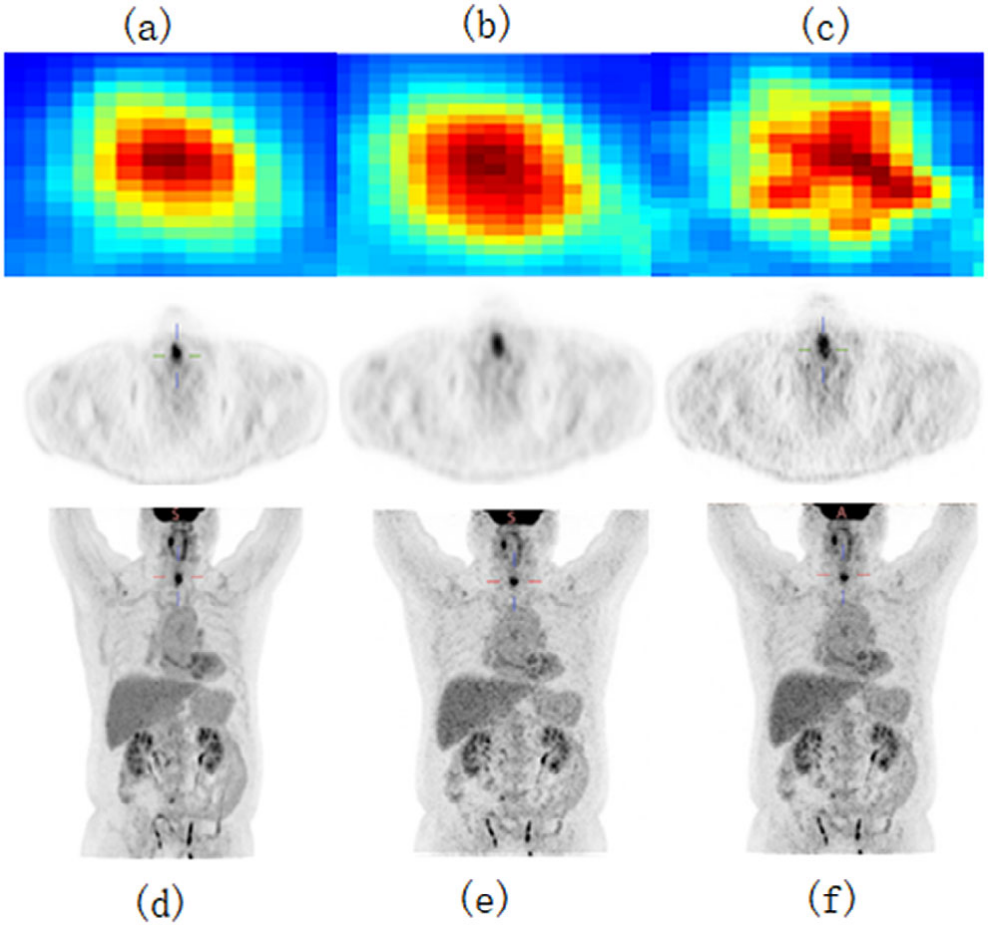
Visualization of standard‐dose images (a, d), Restored images that were generated using our method on the low‐dose images (b, e), Visualization of low‐dose images (c, f).

To comprehensively visualize the experimental results, we selected two representative cases from the test set: As shown in Figures 4 and 5, the contents of Figures 4 and 5 are as follows: Visualization of low‐dose images (a, d), Restored images that were generated using our method on the low‐dose images (b, e), Visualization of standard‐dose images (c, f). the low‐dose PET images exhibit a progressive alignment of lesion contours and shapes towards those in the standard‐dose PET images. Across the test set, replacing standard convolutional layers with residual blocks substantially improved image quality, particularly in terms of detail retention and contour clarity. In summary, incorporating residual blocks enhanced the network's capability to recover high‐quality images, yielding clearer lesion boundaries and improved detail fidelity. These findings underscore the importance of both network architecture and loss function selection in optimizing the quality of low‐dose PET images.

**FIGURE 6:**
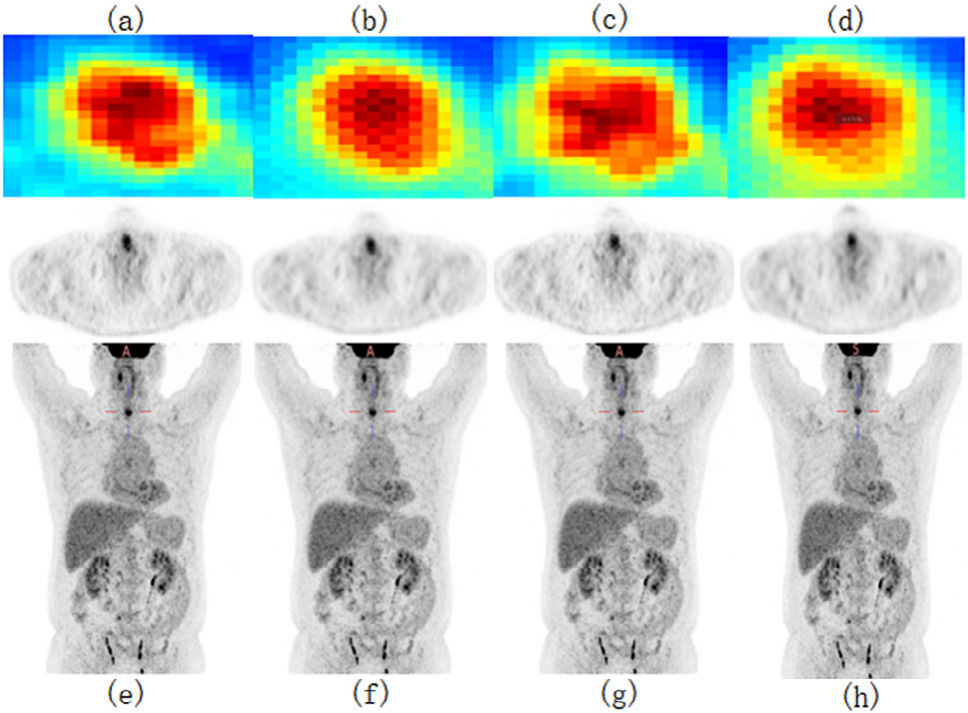
Results of U‐cycleGAN at different downsampling levels: (a, e) four times downsampling, (b, f) five times downsampling, (c, g) six times downsampling; Restored images that were generated using RED‐CNN on the low‐dose images (d, h).

### 3.3 Ablation study

To validate the rationality of the network architecture, we conducted a systematic ablation study, primarily focusing on the number of downsampling operations in the generator and the introduction of residual blocks. First, we evaluated the network's ability to capture local features at different spatial scales by performing varying numbers of downsampling operations on the UET model. We conducted experiments with downsampling from 3 to 8 times, ultimately reducing the feature map size to 1 × 1. The impact of different downsampling rates on the experimental results varied, with some configurations leading to confusion in background colors. The results indicated that an appropriate number of downsampling operations could effectively enhance the network's local spatial processing capability, thereby improving the generation outcomes. This study specifically compared the image quality evaluation metrics of U‐cycleGAN, U‐cycleWGAN, and UR‐cycleWGAN at 4, 5, and 6 downsampling operations, as detailed in Table 3. Compared to most UNet generators within CycleGAN frameworks, our network utilized more downsampling operations, resulting in smaller feature map sizes. The experimental findings demonstrated that five downsampling operations achieved optimal performance in the denoising task, attributed to the inclusion of the perceptual loss function and residual blocks. The residual blocks effectively alleviated the issue of detail loss in images caused by excessive downsampling in the UNet architecture. Additionally, by calculating the differences in high‐level feature spaces, the perceptual loss improved the visual quality and detail retention of the images. The introduction of residual blocks also allowed the network to learn the differences between inputs and outputs directly, mitigating the vanishing gradient problem, thereby enhancing the stability of training in deep networks and enabling better reconstruction of high‐resolution image details despite multiple downsampling operations.

Furthermore, a significant improvement in our network is the direct concatenation of the input image with intermediate feature maps within the network.^38^ This approach greatly enhances the feature fusion capability. During the decoding phase, by combining the original input with the progressively extracted feature maps, the network effectively preserves detail information in the input images, especially excelling in capturing fine structures and edge features. Although this concatenation method increases computational complexity and memory overhead to some extent, it significantly improves the quality of generated images in reconstruction and generation tasks, particularly in terms of detail fidelity. While concatenation may introduce some feature redundancy, its advantages in high‐precision tasks are noteworthy, demonstrating substantial practical value.

**FIGURE 9:**
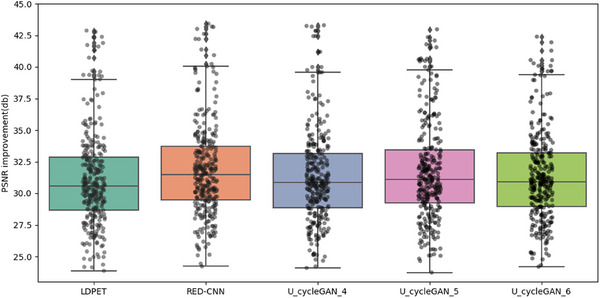
Comparison of PSNR performance for RED‐CNN and U‐cycleGAN at varying downsampling levels, “u_cycleGAN_4″ refers to the results obtained using the u‐cycleGAN model with four downsampling operations.

**FIGURE 10:**
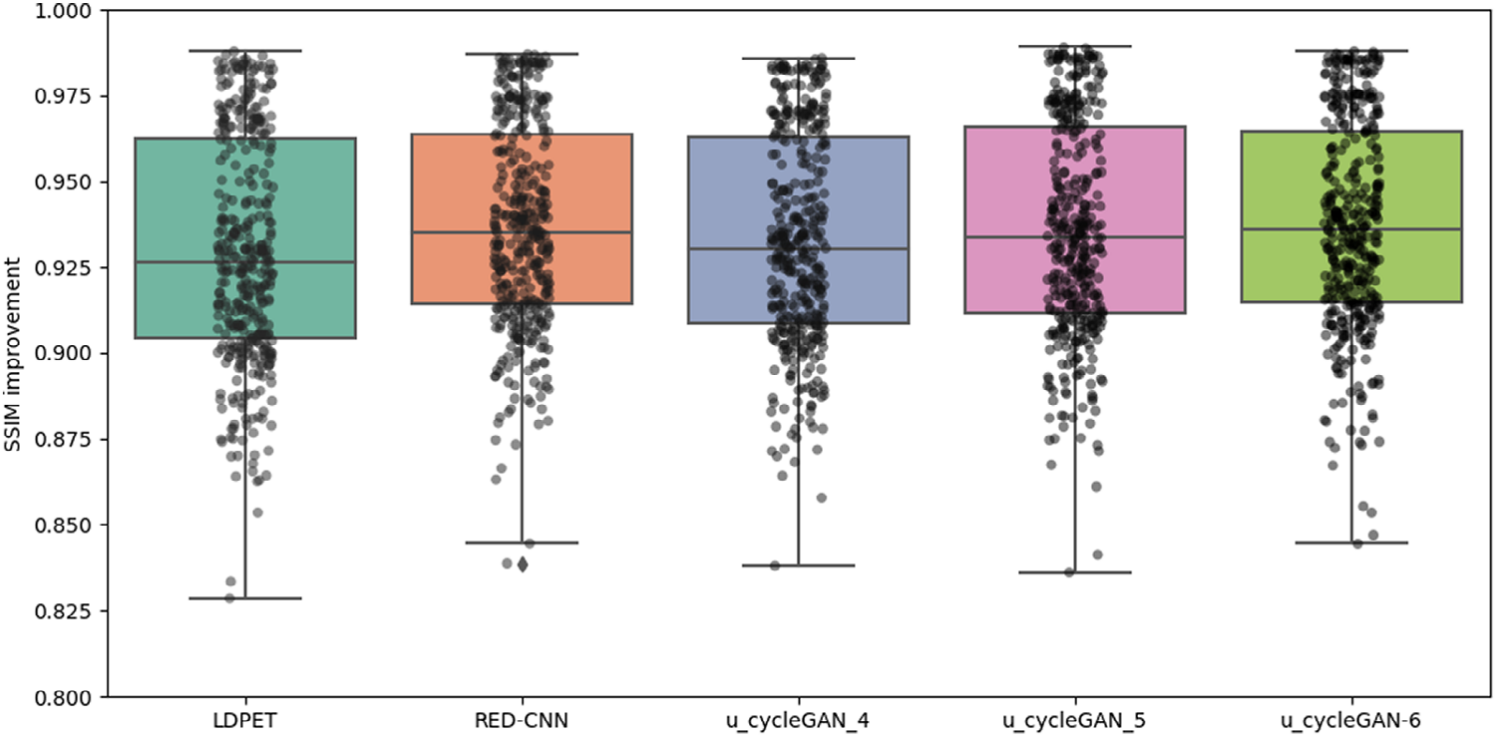
Comparison of PSNR performance for RED‐CNN and U‐cycleGAN at varying downsampling levels.

**FIGURE 11:**
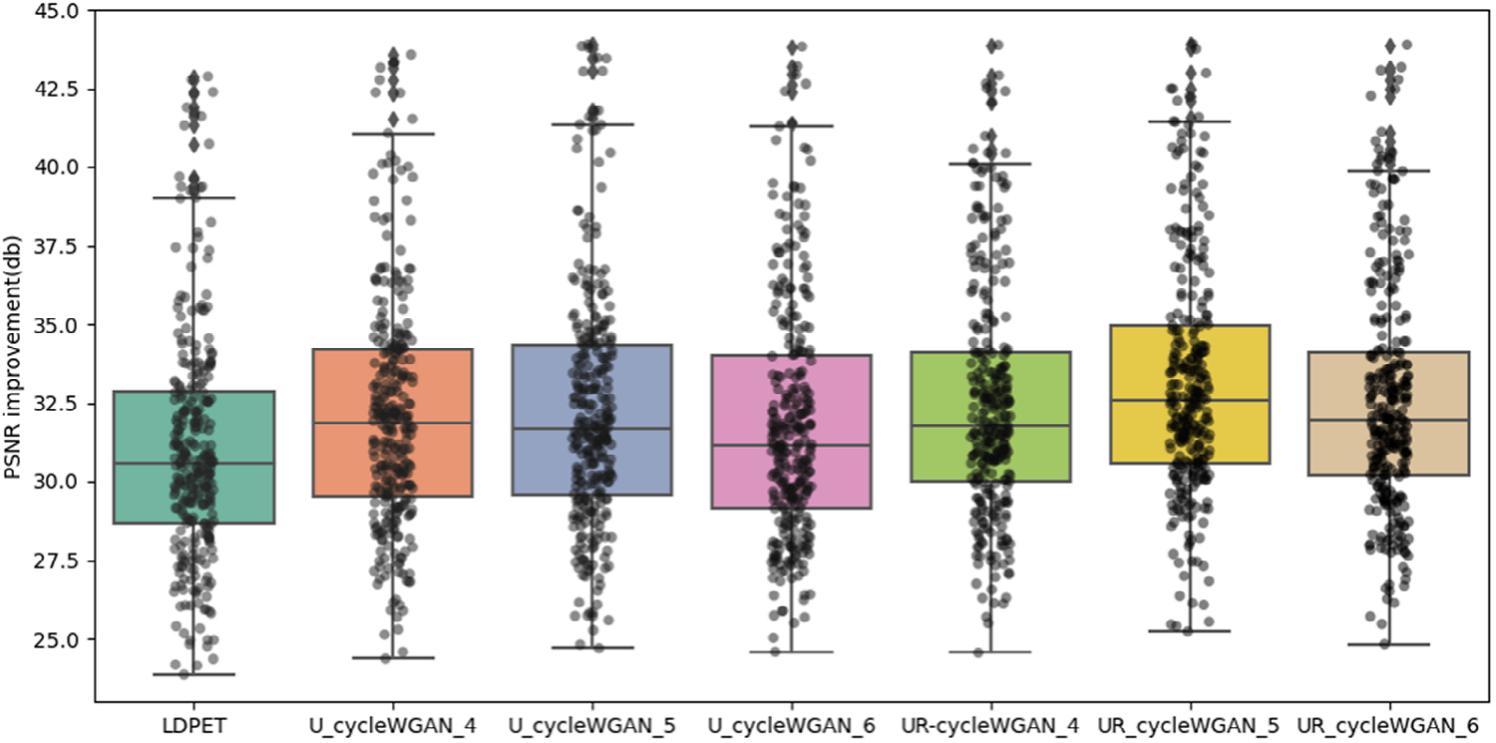
Comparison of PSNR performance for U‐cycleWGAN and UR‐cycleWGAN at varying downsampling levels, “u_cycleWGAN_4″ refers to the results obtained using the u‐cycleWGAN model with four downsampling operations.

**FIGURE 12:**
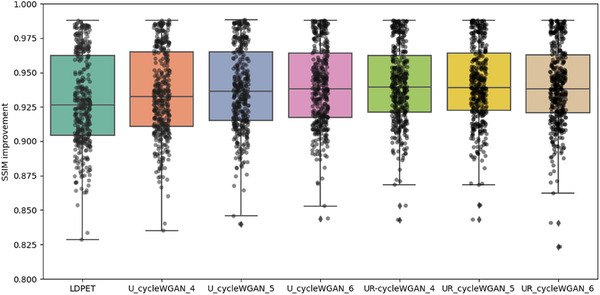
Comparison of SSIM performance for U‐cycleWGAN and UR‐cycleWGAN at varying downsampling levels.

In summary, this study compares U‐cycleGAN, U‐cycleWGAN, and UR‐cycleWGAN on the same dataset. These ablation experiments thoroughly validate the rationality and effectiveness of our network architecture design. We also calculated the average PSNR and SSIM for the entire test set, revealing that each method achieved a certain level of noise suppression. Specific objective analysis metrics can be found in Table 3, as well as Figures 9, 10, 11, 12.

## 4 DISCUSSION

This study evaluates the performance of the proposed deep learning model on low‐dose PET images. The low‐dose PET images were obtained by shortening the acquisition time, with PET data collected at 30 and 150 s per bed position, respectively. All images were acquired during the same scanning session, and the same reconstruction parameters were used for all PET images. In the most related studies, random sampling methods are typically used, where low‐dose images are downsampled from event data (list‐mode format) from SDPET scans. In contrast to using short time frames for simulation, the downsampling method generated LDPET images through separate acquisitions. Due to the differences in the dynamic characteristics of tracers during the imaging process, the underlying signals of LDPET and SDPET images may differ. Additionally, to minimize the potential increase in complexity caused by patient motion during standard acquisition times when estimating SDPET images from LDPET images, this study extracted data during the same session.^39^ Furthermore, because the short time frame PET image reconstruction was performed under the condition where the activity concentration within the acquisition segment was equal to that of the standard PET scan, the number of recorded random events increased significantly, and the random rate is squared in relation to the injection dose. Therefore, the noise level in PET images reconstructed from short time frames will be higher than that in equivalent real or simulated (downsampled) low‐dose scans.^40^

The aim of this study is to develop a technique that uses lower doses to reduce the risk of secondary malignancies in patients undergoing PET/CT examinations with radiation exposure or using a shorter duration to help patients prone to motion artifacts and unable to tolerate longer examinations, while maintaining image quality and diagnostic efficiency, providing new insights into the clinic. This is particularly important for patients who require repeated PET/CT scans throughout the diagnosis and treatment process, but also for pediatric patients.

**FIGURE 7:**
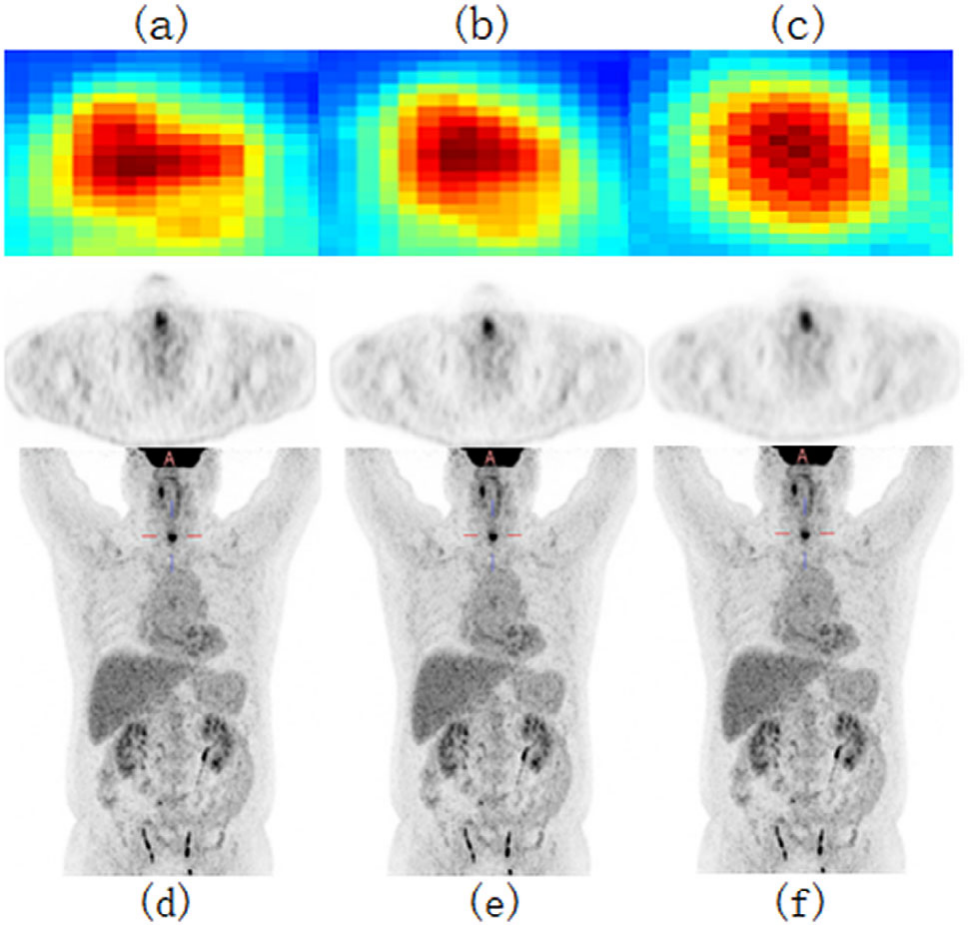
Results of U‐cycleGAN at different downsampling levels: (a, d) four times downsampling, (b, e) five times downsampling, (c, f) six times downsampling.

**FIGURE 8:**
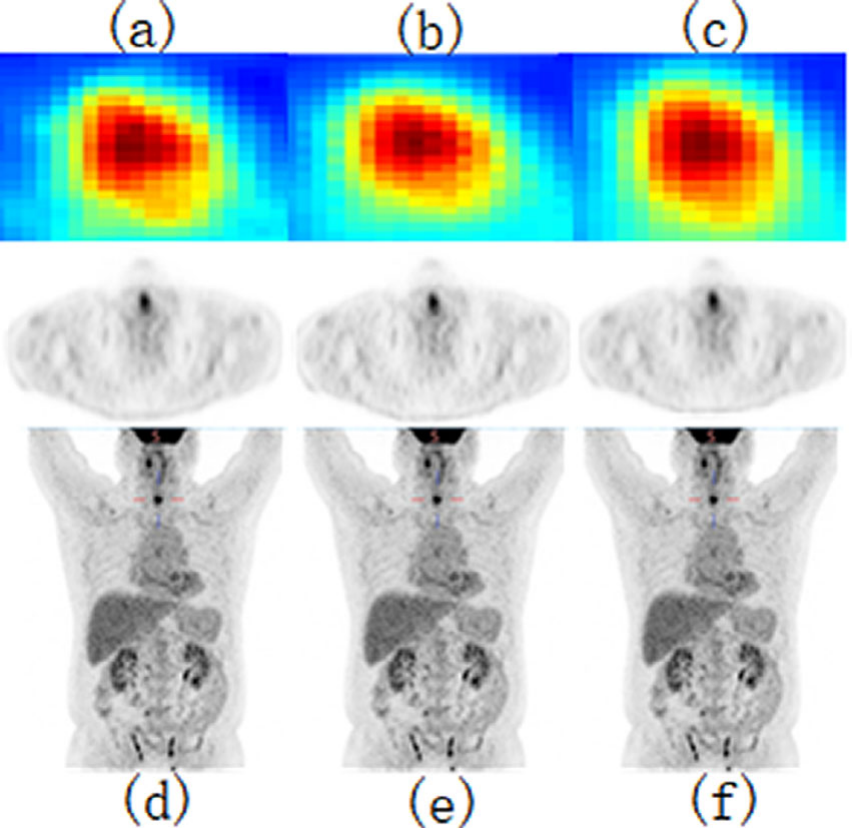
Results of U‐cycleGAN at different downsampling levels: (a, d) four times downsampling, (b, e) five times downsampling, (c, f) six times downsampling.

One of the main innovations of this study is the introduction of the Residual Block into the Deep Learning model. The Residual Block, through its skip connection design, improves the efficiency of information propagation in deep networks and effectively addresses the common gradient vanishing problem encountered during the training of deep networks. The introduction of the Residual Block enables the model to perform feature learning at deeper levels, thereby further enhancing the network's representational capacity. The network is able to better preserve the key information in LDPET while effectively reducing the noise introduced during the LDPET process, thus improving the clarity and detail of the images. In terms of objective image evaluation metrics, our experimental results show that the UR‐cycleWGAN with the introduction of the Residual Block has improved in several important assessment metrics. Specifically, the PSNR and SSIM of the images have significantly increased, indicating effective improvement in image quality. Moreover, in terms of the trend of LESION SUV, the results using UR‐cycleWGAN are closer to those of SDPET. This demonstrates that the Residual Block helps the network better learn the detailed features of SDPET, thereby achieving image recovery results that are more consistent with SDPET. The comparison in Figures 7 and 8 can fully illustrate the effect of the introduction of the Residual Block.

The above experiments demonstrate the overall potential of our method, but there are still some issues worth further discussion. Future work will require more extensive research, including larger‐scale groups and multi‐center, external validation studies, to validate the proposed method.

## 5 CONCLUSION

Our experiments focused on the impact of downsampling layers and the introduction of residual blocks in the U‐Net architecture to validate whether these factors influence the denoising performance of the network. From the experimental results, we observed that performing five downsampling operations on the 256 × 256 original image to generate an 8 × 8 feature map yielded the best results. Notably, our network achieved the smallest feature map size in this domain, demonstrating that even with increased network depth, good denoising performance can still be achieved.

With the advancement of GANs, more well‐structured GAN architectures have emerged. Therefore, there is still considerable room for development in denoising methods for low‐dose PET imaging. CycleGAN, as a reversible neural network, addresses the mapping problem between standard‐dose PET images and their corresponding low‐dose PET images. However, based on the existing data, our network currently uses only a single sequence of low‐dose data and its corresponding standard‐dose data for each bed. If the network could be adapted to a “multi‐input, single‐output” model, it would allow the relationships between different inputs to be connected, providing intermediate state variations. This multi‐input structure would better guide the network in performing low‐dose denoising. This task is an ongoing area of development in our research.

## AUTHOR CONTRIBUTIONS

Yang Liu processed data, write the manuscript, lead the development of proposed algorithm. ZhiWu Sun developed the algorithm, write the manuscript. HaoJia Liu collected the data, conducted the clinical evaluation.

## CONFLICT OF INTEREST STATEMENT

The authors declare no conflicts of interest.

## ETHICS STATEMENT

This study was approved by The Fifth Affiliated Hospital of Zhengzhou University's ethics committee, and patient consent was waived due to the retrospective nature of the data analysis and the use of anonymized data.

## Data Availability

The datasets generated and analyzed during the current study are not publicly available but are available from the corresponding author on reasonable request.
